# The Pain System in Oesophageal Disorders: Mechanisms, Clinical Characteristics, and Treatment

**DOI:** 10.1155/2011/910420

**Published:** 2011-08-02

**Authors:** Christian Lottrup, Søren Schou Olesen, Asbjørn Mohr Drewes

**Affiliations:** ^1^Mech-Sense, Department of Gastroenterology and Hepatology, Aalborg Hospital, Aarhus University Hospital, 9000 Aalborg, Denmark; ^2^Center for Sensory-Motor Interaction (SMI), Department of Health Science and Technology, Aalborg University, 9220 Aalborg East, Denmark

## Abstract

Pain is common in gastroenterology. This review aims at giving an overview of pain mechanisms, clinical features, and treatment options in oesophageal disorders. The oesophagus has sensory receptors specific for different stimuli. Painful stimuli are encoded by nociceptors and communicated via afferent nerves to the central nervous system. The pain stimulus is further processed and modulated in specific pain centres in the brain, which may undergo plastic alterations. Hence, tissue inflammation and long-term exposure to pain can cause sensitisation and hypersensitivity. Oesophageal sensitivity can be evaluated ,for example, with the oesophageal multimodal probe. Treatment should target the cause of the patient's symptoms. In gastro-oesophageal reflux diseases, proton pump inhibitors are the primary treatment option, surgery being reserved for patients with severe disease resistant to drug therapy. Functional oesophageal disorders are treated with analgesics, antidepressants, and psychological therapy. Lifestyle changes are another option with less documentation.

## 1. Introduction

Pain and gastrointestinal (GI) discomfort are the leading symptoms in the multitude of oesophageal disorders. In oesophageal disorders, this review aims to (1) give an overview of the mechanisms underlying abnormal pain processing, (2) describe the clinical features of the most relevant entities, and (3) give a summary of currently recommended treatments. 

## 2. Background

### 2.1. The Nociceptor and First-Order Neuron

**Figure 1:**
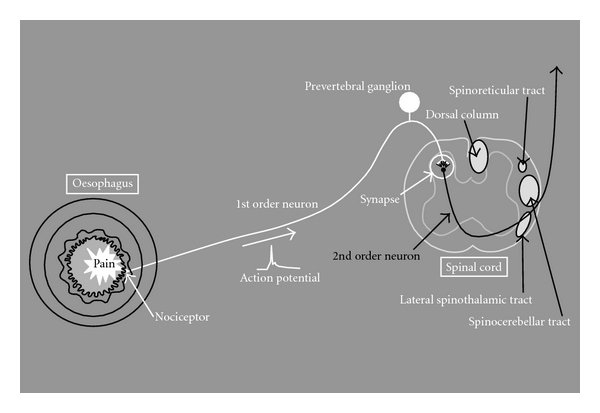
The anatomy and physiology of the pain stimulus. Pain is sensed in a nociceptor in the tissue, which generates an action potential along the nerve fibre of the first-order neuron. The action potential travels proximally towards the other end of the neuron and is transmitted to the second-order neuron in the spinal cord through a synapse mediated by neurotransmitters. The second-order neuron then generates an action potential that travels further proximally through distinct nerve bundles (fasciculi) in the spinal cord and synapses to the third-order neuron in the brain.

Pain is defined as “an unpleasant sensory and emotional experience associated with actual or potential tissue damage or described in terms of such damage” [1]. Generally, noxious stimuli are encoded by receptors (nociceptors) located in the organs. When the nociceptor receives a noxious stimulus strong enough to cause a depolarization, an action potential is generated and transmitted along the first-order neuron to the dorsal horn of the spinal cord (Figure 1) [2].

Unlike cutaneous pain, most visceral nociceptors are probably nonspecific (*polymodal*) and respond to different stimuli being, for example, mechanical, chemical, and ischemic [3]. This information is mainly based on animal studies. In humans, however, the oesophagus has also been described to respond more or less *to specific stimuli* such as electrical, mechanical, thermal, or chemical stimulation [4, 5]. Animal experiments also show that afferent nerve fibres may respond to either *phasic or tonic* distension of the gut [6, 7], and this has to some degree been confirmed in human studies [8, 9]. Some fibres—primarily the mucosal—are *adapting* to a given stimulus and give no response when the stimulus is maintained, whereas afferents in the muscular layers generally show less adaptation [6]. 

The principal *temperature receptors* in the oesophagus are members of the transient receptor potential family. Several temperature receptors exist, among others the transient receptor potential vanilloid 1 (TRPV1), whose threshold for activation can be lowered by hydrogen ions and inflammatory mediators [10]. Acid-sensitive receptors in the gut consist mainly of three groups: TRPV1 (which is temperature- as well as acid-sensitive), acid-sensing ion channels (ASICs), and purino receptors [11]. Acid sensing is important in subgroups of gastro-oesophageal reflux disease (GORD) patients. For example, patients with nonerosive reflux disease (NERD) and erosive reflux disease (ERD) are hypersensitive, whereas patients with Barrett's oesophagus (BO) are hyposensitive to acid (see later sections) [12–15].

The afferent fibres of the first order neuron are either nonmyelinated (70–90%) or thinly myelinated fibres. In organs such as the pancreas and ureter the afferents convey only pain, whereas in others (oesophagus, stomach and rectum) the afferents mediate pain together with other sensations [7, 16]. The visceral afferents mediating conscious sensations run predominantly together with nerves belonging to the sympathetic nerves to reach the CNS, although some afferents join parasympathetic and other pathways [17]. 

### 2.2. Organisation in the Spinal Cord

**Figure 2:**
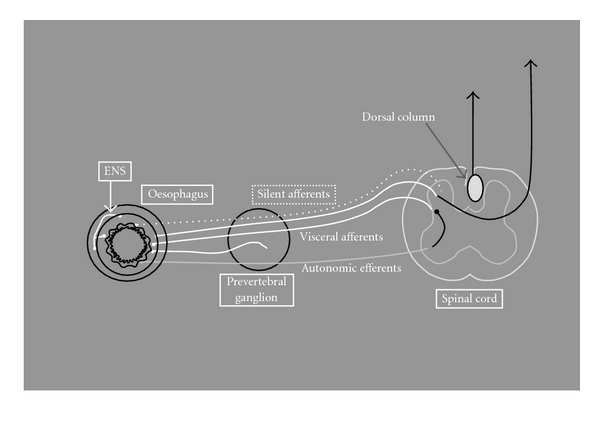
The afferent nerve supply of the gut. “True” visceral afferents (white lines) innervate the gut and run temporarily together with either the sympathetic or the parasympathetic nerves. During inflammation “silent afferents” (dashed line) may become activated. Gastrointestinal (GI) afferents primarily project to the spinal cord or brain stem but can also “crosstalk” with the autonomic (grey line) or enteric nervous system (ENS) through local or spinal reflexes.

All the afferent nerves projecting to the spinal cord terminate in the dorsal horn. From here, the stimulus transmits cephalad through the spinal cord pathways and synapses to the third-order neuron in the brain or brainstem [18]. There is a close interaction and crosstalk between GI afferents and those from the somatic, autonomic, and enteric nervous systems (Figure 2) [19]. Hence, the clinical impression of the patient can easily be changed by complaints such as sweating and palpitations related to the autonomic nervous system as well as symptoms related to somatic referred pain (see later sections). A partial explanation for this is the different organisation of afferents innervating the organs as compared with the somatic nervous system, see next section. 

**Figure 3:**
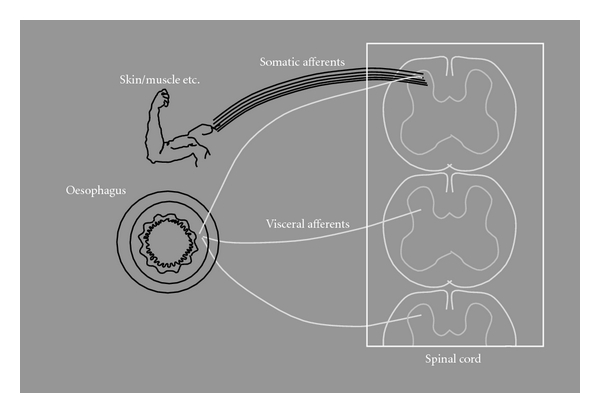
Visceral versus somatic innervation. The relatively low number of visceral afferents terminate diffusely along several segments of the spinal cord, whereas the high number of somatic afferents terminate at one particular level. This is the reason for the diffuse localisation of visceral pain.

The activity in the GI organs does not normally reach the higher brain centres, except from information due to filling of the oesophagus, stomach, and rectum. When the organs are potentially in danger, for example due to diseases, symptoms such as discomfort and pain are sensed. These symptoms are typically vague and difficult to characterise and are often quite distinct from what is felt during noxious diseases of the somatic system. The difference in the anatomical structure of the two nervous systems explains why GI pain is different from the somatic counterpart. The visceral afferents are relatively few and terminate diffusely along several segments of the spinal cord, whereas the relatively numerous somatic afferents terminate at one particular level (Figure 3) [18]. 

**Figure 4:**
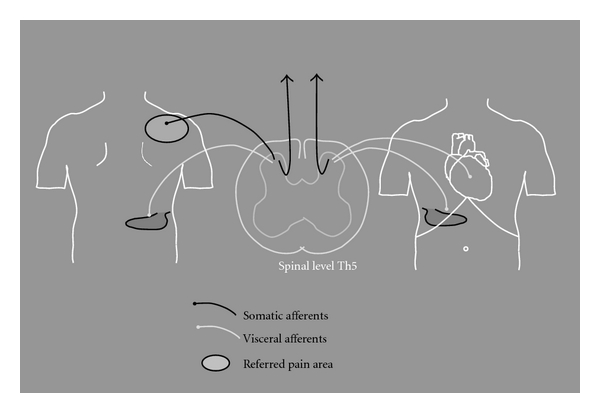
Left: viscerosomatic hyperalgesia: each segment in the spinal cord receives afferent fibres from visceral as well as somatic structures. viscero-somatic hyperalgesia or referred pain originates because of this convergence of spinal afferents. The brain therefore interprets the pain as originating from a somatic structure with the same segmental innervation, for example, referred pain in the right shoulder because of pain originating from the gall bladder. Referred pain can be enhanced by spinal hyperexcitability caused by, for example, local inflammation, hence causing a larger referred pain area. Right: viscero-visceral hyperalgesia: the convergence of visceral afferent nerves in the spinal dorsal horn from different organs results in an increased nociceptive input to this particular segment of the spinal cord. Here shown with convergence of afferent nerves from the heart and gall bladder at the spinal level of Th5. This generates a stronger pain stimulus, a mechanism known as viscero-visceral hyperalgesia.

The fact that each segment in the spinal cord receives afferent fibres from visceral as well as somatic structures causes another phenomenon known as referred pain. Although simplified, referred pain originates due to convergence between visceral and somatic fibres on the same second order (spinal) neuron (for details see [20]). Since the brain cannot localise the precise origin of the visceral pain stimulus, it may therefore interpret the pain as originating from a somatic structure with the same segmental innervation (Figure 4). 

### 2.3. Innervation of the Oesophagus

**Figure 5:**
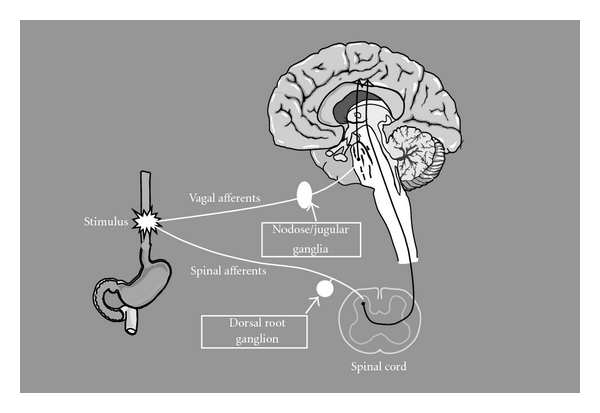
Dual innervation of the oesophagus. The oesophagus is dually innervated by the vagal and spinal nerves. The spinal nerves enter the central nervous system (CNS) through the dorsal root ganglion of the spinal cord from C1 to L2. The vagal afferents travel with the main branch of the vagal nerve, primarily entering the CNS via the nodose and jugular ganglia of the vagal nerve and synapsing in the nucleus of the solitary tract.

The oesophagus is dually innervated by vagal and spinal nerves (Figure 5). Most afferent fibres run temporarily together with either the sympathetic or parasympathetic nerves to enter the spinal cord. Afferents mediating sensory information travel mainly together with the sympathetic nerves, whereas those traveling together with the parasympathetic nerves are mainly involved in secretion, motor, and regulatory reflexes that do not reach consciousness [17]. In the oesophagus, however, nerves traveling together with the vagal nerves also transmit pain [21], and there is increasing evidence for pain transmission via vagal afferents from other segments of the gut [22]. These vagal afferents have their cell body and first synapse located in the nodose and jugular ganglia, from where the second-order neurons project centrally to synapses in the nucleus of the solitary tract in the brain stem [23]. The vagal afferents are able to exert both inhibitory and excitatory influences on spinal nociceptive transmission and modify the conscious sensation [24]. 

The enteric nervous system (ENS) is present in most of the GI tract including the oesophagus. It is closely related to the central nervous system (CNS) and the autonomic nervous system. The ENS is a local minibrain with reflexes and a library of information for different patterns of gut behaviour (Figure 2) [25]. The major functions are control of fluid and electrolyte transport, motility, and reflexes. The ENS is considered a subdivision of the autonomic nervous system [26]. However, unlike other autonomic ganglia that are mainly relay stations, the ENS has mechanisms for independent processing of information like the CNS. Hence, it is working with three functional categories of neurons identified as sensory, inter, and motor neurons. Vagal, but also sympathetic nerves are able to modulate the functions of the ENS via neural synapses, and control of the sensory receptors [26–29]. Furthermore, motility changes are frequently seen during anxiety and stress [26, 30]. 

### 2.4. Central Pain Processing

From the spinal cord, pain transmits to the brain through several distinct pathways (Figure 1). Most afferents travel in the spinothalamic tract to the thalamus. From the thalamus projections to the insula, hypothalamus, and amygdala as well as to higher cortical levels such as cingulate and prefrontal cortices have been described [18, 31]. Insula has an important function for integrating the visceral sensory and motor activity together with limbic integration and is particularly important in pain perception from the gut [32]. The anterior cingulate cortices and prefrontal cortices are a part of the medial pain system, which mediates the affective, emotional, and cognitive components of the pain experience [31, 33]. In addition to the spinothalamic tract, some afferents ascend in the spinoreticular tract mediating arousal and autonomic responses through interaction with the reticular formation. Finally, afferents ascend in the spinomesencephalic tract, which relates to a complex neuronal network in the brain stem involved in endogenous pain modulation [18].

The pain system has several inherent mechanisms whereby inflowing pain signals can be modulated. The multiple complex pathways involved in this modulation comprise both spinal and supraspinal regions. In particular, connections between these sites, commonly referred to as the descending modulatory pain pathways, play a central role [34, 35]. Pain modulation can lead to either an increase in the transmission of pain impulses (facilitation) or a decrease in transmission (inhibition). The balance between these states ultimately determines the quality and strength of pain signals leading to pain perception in the brain. Alterations in the state of descending modulatory pathways towards facilitation have been associated with the transition of acute into chronic pain [36]. 

### 2.5. Sensitisation

#### 2.5.1. Peripheral Sensitisation

*Peripheral* nociceptor sensitisation underlies the hyperalgesia that develops immediately around an injury site. Analogous to mechanisms documented in the cutaneous system, oesophageal afferent fibres may become sensitised by endogenous chemicals. This results in an increase in their responsiveness to a given stimulus and/or an increase in the spontaneous activity [37]. In contrast to the cutaneous system, where only nociceptors sensitise, both low- and high-threshold fibres in the viscera can undergo sensitisation [22, 38]. Upon local oesophageal inflammation, caused by, for example, acidic reflux, various inflammatory mediators including protons, prostaglandins, serotonin, and histamine are released. These reduce the perception threshold of primary afferents and recruit previously silent nociceptors (Figure 2). This again leads to increased afferent activity to the spinal cord and exacerbation of the pain [39]. Furthermore, an upregulated expression of nociceptors such as sodium channels, TRPV1, ASICs, and purino receptors are seen during inflammation. As a consequence of all of these changes, the pain sensitivity at the site of inflammation is increased [40, 41]. In human experimental studies, sensitisation of the oesophagus has been induced by mucosal perfusion of hydrochloric acid and capsaicin [5, 42, 43]. 

#### 2.5.2. Central Sensitisation

Enhanced spinal input from, for example, oesophageal inflammation can activate intracellular signalling cascades within the spinal dorsal horn neurons. This results in an increased synaptic efficacy and is known as central sensitisation [39]. Deep pain and visceral pain input to the spinal cord are more potent than cutaneous pain in the induction of central sensitisation [39]. Simplified, the input leads to the activation of the N-methyl-D-aspartic acid receptor and results in changes of the resting potential of the second-order neuron [18]. It is thus rendered more likely to fire when stimulated by the chemical transmitters. This is thought to be a major factor of central sensitisation [44]. Blocking the N-methyl-D-aspartic acid receptor has been shown to prevent experimentally acid-induced central sensitisation [44]. This effect is used clinically, where antagonism of the receptor during operations leads to less postoperative pain [45]. 

#### 2.5.3. Viscero-Visceral Hyperalgesia

Viscero-visceral hyperalgesia is a complex form of hypersensitivity probably explained by more than one mechanism. Since this phenomenon takes place between visceral organs which share their central afferent termination, it is plausible that central sensitisation play an important role [46]. Animal experiments as well as human experimental studies have shown that convergence to the same second order neuron of afferents from different organs exists [43, 47–49]. Also, our group has shown that oesophageal perfusion with acid and capsaicin increased the sensitivity in the rectum and that the referred pain areas to duodenal stimulation were increased [50–52]. Besides changes at the spinal level, changes in the cortical processing of pain may be involved in these mechanisms [53]. The widespread visceral hypersensitivity in functional GI disorders (irritable bowel syndrome, functional dyspepsia, etc.) may be due to this mechanism [54–56].

Viscero-visceral hyperalgesia may explain the epidemiological findings that several clinical conditions with organic diseases show evidence of increased pain from other organs (Figure 4). This was recently investigated in patients suffering from a combination of either coronary artery disease and gallbladder stones or inflammatory bowel disease and dysmenorrhoea [57]. It was shown that a patient having more coexisting visceral pain conditions with common spinal projection generated more symptoms from the other pain condition. Besides, effective treatment of one condition significantly improved symptoms from the other [57]. 

## 3. Sensation in Oesophageal Disorders

### 3.1. The Oesophageal Multimodal Pain Model

**Figure 6:**
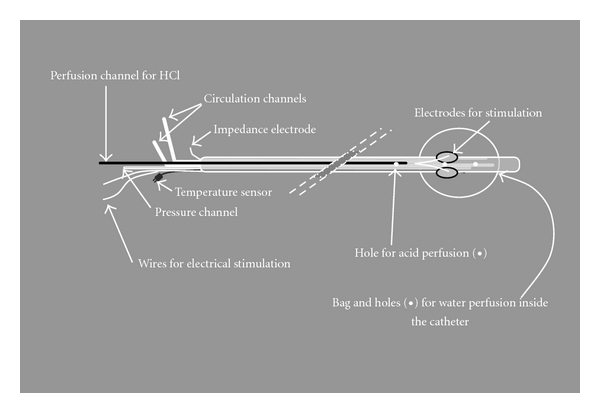
The multimodal probe. The probe is placed in the lower oesophagus with the bag located 8 cm above the oesophagogastric junction. The probe can be used to give painful stimuli by mechanical inflation of the bag with saline, giving a weak electrical shock or circulating heated or cold water inside the probe. Hyperalgesia can be evoked with acid and/or capsaicin infusions through a hole proximal to the bag. This way, the oesophageal sensitivity to different pain modalities can be investigated in one procedure.

Most information of visceral pain physiology comes from animal studies. However, human experimental pain models have been extensively used in visceral pain studies to elucidate the sensory properties of the gut, that is, the effects of age, gender, and menstrual cycle on pain as well as differences in the sensitivity of several parts of visceral hollow organs [58–60]. Models including the Barostat based on balloon distension, the Bernstein test (in which acid or saline is perfused in the oesophagus), and electrical stimulation of the oesophagus have been used for many years [58, 61]. The problem with these models is that only one or two pain modalities can be examined with each model, for example, pain to electrical current and acid. If several modalities are to be examined, it would require multiple intubations, most likely resulting in a dropout of volunteers. The multimodal pain model was developed to overcome these obstacles. With one catheter and one intubation only, it is possible to stimulate the oesophagus with distension, cold, heat, electrical current, and liquid chemicals (Figure 6) [16]. The main advantage of the model is that it allows a differentiated assessment of the superficial and deep structures of the gut wall, activation of different nerve fibres, and peripheral as well as central pain mechanisms. The model is reproducible and has been widely used to understand basic sensory mechanisms in the oesophagus as well as mechanical properties. The validity of the model was confirmed in studies where the model was used to explore the pathophysiology of oesophageal disorders such as ERD and NERD, BO, and noncardiac chest pain (NCCP) [12, 62–65]. The findings in these and other studies on oesophageal sensitivity are summarised in Table 1. 

### 3.2. Neurophysiological Assessment

In most experimental studies, assessment of pain has relied on indirect measurements of self-reported pain. These subjective measurements have been improved with the use of validated psychophysical scales like the visual analogue scale, the McGill pain questionnaire, and the Gracely pain scale [19]. As opposed to the subjective assessment of pain, neurophysiological assessment provides an objective way to characterise the pain response. In humans, the brain response to experimental oesophageal pain has been studied using positron emission tomography (PET), functional magnetic resonance imaging (fMRI), and methods based on evoked brain potentials. In PET studies, painful dilatation of the oesophagus was shown to activate mainly the anterior insular cortex and anterior cingulate gyrus [66]. In studies based on fMRI, parallel findings have been reported [66, 67]. 

Although PET and fMRI possess a high spatial resolution (millimetres), they are hampered by a poor temporal resolution (seconds). Thus, the retrieved brain response using these methods corresponds to rather late events that may be related to arousal and attention rather than pain. On the contrary, evoked brain potentials detect neuronal activity in real time with a very high temporal resolution (milliseconds). Using this technique, the sequential brain activity following electrical stimulation of the oesophagus was determined in a study from our group [68]. Furthermore, studies based on advanced evoked potential analysis have shown evidence of changes in central pain processing following electrical stimulation of the oesophagus. The findings have been made in healthy volunteers and in patients suffering from functional chest pain (among others [69], see also later sections). 

## 4. Clinical Aspects of Oesophageal Disorders

The clinical picture of oesophageal disorders depends on the pathophysiology responsible for their development. In some diseases, for example ERD, excess oesophageal acid exposure is thought to be the main pathophysiological factor underlying their development [70]. Hence, ERD is considered an organic disorder which should be cured by removing the main cause of the disease, namely, oesophageal acid exposure. In contrast, functional chest pain (FCP) symptoms are believed to be caused by some kind of oesophageal hypersensitivity without objective pathological findings, that is, a functional disorder as the name suggests [71–74]. 

### 4.1. Nonerosive and Erosive Reflux Disease

GORD is defined as chronic mucosal damage or typical symptoms of reflux disease, which reduce quality of life, combined with the retrograde reflux of gastric contents into the oesophagus [75]. The typical symptoms include heartburn, regurgitation, and/or chest pain; furthermore, many patients consider heartburn a painful feeling. GORD is a very common disorder with up to 30% of the European population reporting heartburn and/or acid regurgitation during the previous 12 months [74].

Usually, GORD is divided into ERD and NERD. When classified according to endoscopy, 24-hour pH profile and symptom-reflux association indices, recent studies have revealed that up to 70% of the GORD patients have NERD. However, the quality of life impairment in patients with NERD is comparable to that in patients with ERD [76]. Since the symptoms in reflux disease are highly variable and poorly understood, no general relation seems to exist in patients with GORD between the symptoms and severity of the disease categorised endoscopically [74, 77, 78]. Adding 24-hour oesophageal impedance monitoring to the pH measurement has given the possibility of identifying subgroups of these patients who have reflux of nonacid or weakly acidic gastric contents [79]. In this way, some patients who were earlier classified as having no significant reflux can now be correctly classified. However, the clinical importance of this is uncertain, especially in NERD patients [80]. 

Conclusively, in many patients the exact cause of symptoms remains unclear. Some patients will, however, fulfil the definition of functional oesophageal disorders, see next section. 

### 4.2. Functional Oesophageal Disorders and Chest Pain

#### 4.2.1. Definition and Prevalence of Functional Disorders

According to the Rome III criteria, FCP is defined as* Midline chest pain or discomfort that is not of burning quality, absence of evidence that gastrooesophageal reflux is the cause of the symptom, and absence of histopathology-based oesophageal motility disorders*. The criteria must be fulfilled for the last 6 months with symptom onset at least 3 months prior to diagnosis [81]. Another group of patients have functional heartburn (FH), for which the Rome III criteria are the same, except that their discomfort or pain is located retrosternally and is described as burning. These patients probably resemble the ERD and NERD patients more than FCP and show better improvement on proton pump inhibitor (PPI) therapy. Functional chest pain has also been termed NCCP and unexplained chest pain, terms that according to the Rome III criteria are somewhat resembling FCP but also include the definition of FH. Due to these similarities and the fact that few studies have examined them separately, FH is included in FCP in this review. Some clinical characteristics of FCP are summarised in Table 2. 

The prevalence of FCP is much debated, and few studies have used the Rome III criteria. With widely varying criteria, the prevalence has been estimated anywhere between 2 [96] and 46% [97]. Furthermore, the disease accounts for approximately 2–5% of presentations to emergency departments and 5% of presentations to primary care physicians [98, 99] and therefore has major implication on any health care system. Before the diagnosis of FCP is established, patients should undergo thorough evaluation (cardiac and gastroenterological) to exclude other diseases causing the pain [99, 100]. 

#### 4.2.2. Possible Mechanisms for Hypersensitivity in Functional Chest Pain

Formerly, oesophageal hypersensitivity to otherwise physiological stimuli was considered a likely origin for FCP. Hence, the disorder has been termed “irritable oesophagus” or “acid-hypersensitive oesophagus” [71–73]. Nowadays, symptoms associated to reflux events are considered a part of the spectrum of symptomatic GORD [100]. In several studies, Rao et al. have reported increased oesophageal sensitivity to balloon distension in patients classified with NCCP [87–89]. However, our group has later performed a similar study using multimodal oesophageal stimulation, applying a protocol better designed to detect the pain response to oesophageal distensions. We did not find hyperalgesia at baseline in NCCP patients but did find lowered pain thresholds to distension and increased referred pain areas after sensitisation with acid perfusion [101]. A recent study similarly found lowered pain thresholds after acid sensitisation in FH patients compared to healthy controls [102]. Since referred pain inevitably involves central mechanisms, this is a strong indicator of central sensitisation in NCCP/FCP/FH patients. This was also proposed by Sarkar et al., who found secondary viscero-visceral and viscero-somatic hypersensitivity to painful stimuli after oesophageal acid perfusion in NCCP patients [48]. Furthermore, several neurophysiological studies have analysed evoked brain potentials after painful oesophageal stimulation [43, 49, 69, 103–105] in NCCP patients. They have found consistent results indicating abnormal central pain processing and sensitisation. Furthermore, others have found the activation of autonomous reflexes, primarily increased vagal tone, following acid stimulation [103, 106]. Besides central sensitisation, peripheral nerve sensitisation is probably present in FCP as well as in other oesophageal pain disorders [107], but few studies in this area have been done. 

### 4.3. Barrett's Oesophagus

Barrett's oesophagus (BO) is a metaplastic condition in the oesophagus, in which the squamous epithelium is replaced with columnar epithelium, so far considered to arise due to excess gastro-oesophageal reflux [108]. Further clinical characteristics are summarised in Table 2. The prevalence is rising and probably around 1.6% in the general population of Western countries [109]. The most important clinical aspect of the disorder is the largely increased risk of oesophageal adenocarcinoma, possibly as much as 125 times compared to the general population [110]. 

#### 4.3.1. Sensitivity in the Oesophagus

Patients with BO are most likely hyposensitive to oesophageal stimuli, but the power of evidence for the hyposensitivity is variable between the different stimulus modalities (Table 1). Several studies have found indications of lowered acid sensitivity in patients with BO. This is true for clinical [13, 84] as well as experimental studies [14, 111, 112]. Using the multimodal pain model, our group investigated the sensitivity in patients with BO. We showed hyposensitivity to mechanical distension in the lower (metaplastic) part of the oesophagus and hyposensitivity to both mechanical and heat stimulation in the normal mucosa in the mid-oesophagus [64]. This could indicate that the sensory abnormalities precede the disease rather than being a consequence of the metaplasia. Furthermore, no indication of central involvement in the oesophageal hyposensitivity was found [64]. Similar results of hyposensitivity in BO to oesophageal balloon distension were found in another study [83]. Hence, a dysfunction of the afferent sensory pathways could be one explanation for hyposensitivity and a possible pathogenetic factor in BO. Potentially, a number of acid receptors could be involved in the hyposensitivity [113]. Among these, the best candidate is the TRPV1 receptor which has previously been shown to be sensitive to heat as well as acid [11, 114]. 

#### 4.3.2. Impaired Acid Clearance

Several indications exist that patients with BO have an impaired ability to clear refluxed acid from the oesophagus. An excess acid exposure on 24-hour pH measurement has previously been shown in BO compared to both healthy controls and other GORD patients [70, 115]. Excess acid exposure in BO could indicate an impaired ability to clear refluxed acid. Furthermore, at least one TRPV1 receptor-mediated oesophageal peristaltic reflex protecting the mucosa from harmful stimuli has been shown to be impaired in patients with BO [116]. Finally, oesophageal motility disorders including lower oesophageal sphincter insufficiency and decreased peristaltic amplitudes have been shown to be overrepresented in patients with BO [117]. These findings could be part of the possible explanation for this impaired acid clearance. 

## 5. Treatment of Oesophageal Pain and Related Symptoms

### 5.1. Acid Suppression

Overall, PPIs are the most effective acid-suppressive drugs available and are the drug of choice for GORD (Table 2). Symptoms such as painful heartburn and isolated chest pain have been shown to respond very fast to treatment with PPIs [93, 118, 119]. 

A meta-analysis by Khan et al. including 35.978 patients with erosive oesophagitis found PPIs to be roughly 4 times as effective as placebo and twice as effective as histamine 2 receptor antagonists in healing erosive oesophagitis [118]. Also, they showed a modest, but significant effect on the healing of oesophagitis when doubling the PPI dose, with a number needed to treat of 25 [118]. However, in the treatment of heartburn symptoms without endoscopic lesions, the effect of PPIs is less convincing. Most studies have included NERD and FCP as a whole, despite the previously mentioned differences between these groups. In a meta-analysis, Hiyama et al. found a significant symptom improvement rate of 68% on PPI treatment in NERD patients, but the results of the included studies were conflicting [93]. In another meta-analysis, Cremonini et al. found PPI treatment to be superior to placebo on symptom improvement in NERD, with a number needed to treat of 3 [119]. On the opposite, Dean et al. concluded that the difference in symptom response rates between PPIs and placebo (the therapeutic gain) was only 27% in NERD (compared to 48% in ERD) [90].

A probably important factor influencing the response to PPI treatment in NERD patients is the degree of distal oesophageal acid exposure measured with 24-hour oesophageal pH. A close relationship was shown between long acid exposure time and the response to PPIs in a large clinical trial [120].

Doubling the dose of PPI using a standard dose twice daily is used in certain cases. In the treatment of ERD, this is supported by reasonable evidence with a NNT of 25 for the doubling of dose [118]. However, when treating NERD, in an attempt to relieve patients not responding to conventional doses of PPI, the evidence supporting the doubling of dose is lacking [91]. Yet some recommendations, including those of the American Gastroenterological Association Institute, recommend trying out double dose of PPI for persistent oesophageal symptoms, regardless of endoscopic findings [121]. 

When considering which PPI to use, price and degree of symptoms must be taken into consideration. The potency of the different PPIs is somewhat varying, but the overall efficacy is good. A meta-analysis by Kirchheiner et al. including 57 clinical studies [95] found relative potencies compared to omeprazole between 0.23 and 1.82 for the currently available PPIs (Table 3). Despite the differences in theoretical potency, no major differences in regard to the clinical effect have been found between the different PPIs [91]. 

When it comes to histamine 2 receptor antagonists, several studies have found these to be superior to placebo (but inferior to PPIs) in healing reflux oesophagitis, including the above-mentioned meta-analysis [118, 122]. Therefore, these drugs remain primarily a supplement to the PPIs but can be used as on add-on to PPI or as sole therapy when minimal symptoms are present or due to economic considerations [91, 123]. Histamine 2 receptor antagonists have also been used in nightly reflux where PPIs are less efficient, although tachyphylaxis limit their long-term use [122].

Conclusively, the primary choice of acid suppressive treatment in patients who fulfil the criteria for GORD, regardless of other characteristics, should be a PPI of any choice in the standard dose. This said, those who have normal oesophageal mucosa on endoscopy (NERD) and especially those with negative 24-hour pH (FCP and FH patients according to the previous definitions) benefit less from acid-suppressive treatment than ERD patients. However, they most likely still have a better response to PPIs than to placebo [91, 92, 121]. Histamine 2 receptor antagonists can also be used as supplement to PPIs or because of the lower price but are less effective. 

### 5.2. Surgery

Surgical treatment of reflux is more effective than medical treatment of reflux and pain. The primary surgical method is Nissen fundoplication, in which the gastric fundus is wrapped around the oesophagus to create an artificial valve to support the insufficient upper oesophageal sphincter. A meta-analysis including 1232 patients showed improvement in quality of life, GORD-related symptoms, and 24-hour pH up to 1 year after an operation with Nissen fundoplication compared to medical treatment with PPIs [124]. However, data on long-term outcome were insufficient, and side effects should also be taken into consideration. Most important side effects in patients treated surgically are dysphagia (6%), risk of reoperation (up to 7% within 3 years), and different abdominal symptoms such as inability to belch, bloating, and diarrhoea (up to 36%) [91, 125]. 

### 5.3. Other Drugs

Transient lower oesophageal sphincter relaxations have been shown to be a major contributor to acidic and nonacidic gastro-oesophageal reflux, especially in NERD patients [126, 127]. Several drugs have been developed to reduce the number of these relaxations. These include gamma amino butyric acid B (GABA_B_) agonists, metabotropic glutamate receptor antagonists and cannabinoid receptor 1 agonists, which, hence, can be used to treat reflux and associated chest pain. So far, only GABA_B_ agonists have proven clinically valuable, whereas the two latter still await clinical studies [127]. Baclofen, a specific GABA_B_ agonist, acts on the central level and has been shown to reduce the rate of transient lower oesophageal sphincter relaxations and increase lower oesophageal sphincter basal tone. Besides, it has proven effective on clinical parameters such as oesophageal acid exposure, nonacid reflux, bile reflux, and symptom improvement [127]. Baclofen has noticeable side effects such as dizziness and nausea, due to the central action. Despite this and a short half life of 3-4 hours, it has been used in the treatment of GORD [128, 129]. Another promising GABA_B_ agonist is lesogaberan, which has a theoretical effect similar to baclofen, but acts primarily in the periphery. So far, lesogaberan has only been investigated in one clinical study in GORD patients, which found significant reduction on the number of transient lower oesophageal sphincter relaxations and reflux episodes and side effects on placebo level [130]. 

### 5.4. Lifestyle Modification

The evidence supporting a beneficial effect of lifestyle interventions for the treatment of oesophageal pain disorders is sparse. A comprehensive review by Kaltenbach et al. found weight loss and head of bed elevation to be the only certain factors to improve the course of GORD [131]. Both were shown to improve symptoms and acid exposure time measured by oesophageal 24-hour pH. In the same review, however, neither smoking, alcohol, nor high-fatty meals showed any effect on clinically measurable variables. This is somewhat surprising, since there is physiological evidence that these three factors do increase the number of transient lower oesophageal sphincter relaxations [131]. 

Weight loss has been found to have a probable positive effect on GORD [131, 132]. However, this was based on symptom assessment from questionnaires, whereas more robust signs of improvement such as oesophageal 24-hours pH-measurement failed to show an effect. Despite the lack of evidence, guidelines, for example the American Gastroenterological Association [121, 122], recommend avoiding late meals, specific foods, alcohol, or specific activities in selected patients, especially in patients who have experienced that this is effective in controlling their symptoms. 

### 5.5. Symptomatic Pain Treatment in Oesophageal Disorders

FCP should not be treated as such before a thorough evaluation has excluded other causes of the patient's symptoms. If a diagnosis of a functional oesophageal disorder is established, the approach to the patient should be based on treatment of symptoms. Patients with heartburn and negative acid exposure have greater anxiety and somatisation scores than those with verified reflux [100]. This means that a psychological approach including explanation of the mechanism behind and reassurance of the diagnosis probably helps the patient to cope better with the symptoms. Psychological intervention in the form of cognitive behavioural therapy undertaken by a psychologist or psychiatrist has been demonstrated effective in patients classified with NCCP in clinical trials [133–135].

When it comes to pharmacological treatment, conventional pain treatment is only one option. Here, the principle of the analgesic ladder applies [136]. This means that paracetamol should be used as first-line analgesic. Nonsteroid anti-inflammatory drugs are the usual second step in pain treatment, but caution should be taken when treating patients with GI diseases because of the risk of bleeding and strictures. If patients have chest pain due to musculoskeletal disorders, NSAIDs are the natural choice. However, in risk groups such as previous ulcer or old age, parallel treatment with a PPI is recommended. If paracetamol is not sufficient and GI disease is present, a weak opioid can be chosen. In other diseases with chronic pain, this has been demonstrated more effective than morphine and with less side effects [137]. 

Other drugs with effect on FCP are tricyclic antidepressants and selective serotonin reuptake inhibitors, which have been demonstrated effective on functional GI disorders including FCP in controlled trials [94, 100, 135, 138]. Drugs developed for epilepsy such as gabapentin and lamotrigine can also be effective in certain cases. On a more experimental level and in an open label study, theophylline was shown to increase the pain threshold to experimental pain in patients with functional chest pain compared to placebo [89]. If a primary motility dysfunction is suspected, treatment with nitrates or calcium channel blockers can be considered [139]. 

### 5.6. Summary of Treatment Recommendations

In summary, when approaching a patient with reflux symptoms suspected for GORD, the general strategy is to start with a short course (e.g. 4 weeks) of PPI in the standard dose as a diagnostic test. This has been shown to be the most cost-effective and time-saving approach with a good sensitivity as well as specificity in diagnosing NERD [99, 119, 140]. If the patient responds, the PPI dose is tapered to the lowest dose relieving the symptoms. In case of treatment failure, other evaluation such as endoscopy, oesophageal 24-hour pH-impedance monitoring, and high-resolution oesophageal manometry should be undertaken. Depending on the results of these tests, treatment adjustment can be considered. Surgery should probably be reserved for younger patients deeply troubled by their symptoms despite high-dose PPI treatment, and only after careful evaluation has concluded that gastro-oesophageal reflux is the true cause of the symptoms. Lifestyle modifications can always be considered, but recommendations should be individualised since no parameter is overall effective. In functional oesophageal disorders, psychological therapy supplemented by pain treatment with analgesics should be the choice. 

## 6. Conclusion

Disorders of the oesophagus show heterogeneous characteristics in relation to the pathogenesis and symptoms and therefore also require an appropriate investigation and a treatment adjusted to the findings. Methods like the multimodal model for oesophageal pain characterisation have been developed to supplement the conventional investigations such as oesophageal 24-hour pH and impedance measurement and manometry. Furthermore, neurophysiological assessment such as PET, fMRI, and analysis of evoked brain potentials can potentially contribute to the clinical evaluation of these complex disorders in the future. Treatment should be directed against the cause of the disease. When for example, gastro-oesophageal reflux is the cause of symptoms, the recommended treatment is acid suppression with primarily PPIs and in rare cases surgery. Handling of functional oesophageal disorders should include acid suppression and analgesics but also consider anti-depressants and psychological assessment and treatment. Lifestyle changes as treatment of oesophageal disorders can also be tried but should be directed according to the individual effect on symptoms in each patient. 

## Figures and Tables

**Table 1:** Sensitivity change to different stimuli in various oesophageal disorders relative to healthy controls.

Disorder	Acid and/or capsaicin	Distension	Heat	Central sensitisation
ERD	↑ [15]	↓ [82]	↑ [10]	↑ [10]
NERD	↑ [12]; ↑ in pH−,→ in pH+ [83]	↓ [12]	↑[12]	↑ [12]
Barrett's oesophagus	↓ [83–85]	↓ [64]	↓[64]	→ [64]
FCP/FH	↑↑ [86]	↑ [87–89]	?	↑ [48, 65]
Eosinophilic oesophagitis	↑ [62]	→ [62]	→[62]	→ [62]

↑: increased; ↑↑: highly increased; ↓: decreased; →: no change; ?: insufficient data; ERD: erosive reflux disease; NERD: nonerosive reflux disease; FCP: functional chest pain; FH: functional heartburn; pH−/+: negative/positive 24-hour oesophageal pH monitoring (positive if time with pH < 4 is above 5%).

**Table 2:** Clinical characteristics of different reflux disorders.

Disease	Visible lesions on endoscopy	24 h pH	Typical symptoms	Symptom response to PPI	Symptom response to placebo
ERD	Yes (erosions)	Positive	Heartburn	Very good (app. 60 to 80%) [90, 91]	Very poor (app. 10%) [90, 92]
NERD	No	Variable	Heartburn	Intermediate to good (app. 40% [90] to 70% [93])	Very poor (app. 15%) [90, 92]*
Barrett's oesophagus	Yes (Barrett mucosa)	Positive	Heartburn, none	Intermediate	?
FCP/FH	No	Negative	Heartburn, chest pain	Intermediate (app. 50%) [94]	Very poor (app. 5%) [94]

*Including FCP; 24-hour pH: typical results of 24-hour oesophageal pH monitoring (positive if time with pH < 4 is above 5%); PPI: proton pump inhibitor; app.: approximately; ERD: erosive reflux disease; NERD: nonerosive reflux disease; FCP: functional chest pain; FH: functional heartburn; ?: insufficient data.

**Table 3:** Potency of different PPIs as measured by effect on mean intragastric 24-hour pH. Figures from [95].

PPI	Relative potency compared to omeprazole
Rabeprazole	1.82
Esomeprazole	1.60
Omeprazole	1.00
Lansoprazole	0.90
Pantoprazole	0.23
